# Comparison of a Telehealth-Based Intensive Treatment Program with a Rewarding App vs. On-Site Care for Youth with Obesity: A Historical Cohort Study

**DOI:** 10.3390/children10071117

**Published:** 2023-06-27

**Authors:** Khen Sela Peremen, Shay Maor, Amit Yaniv, Ishai Aloni, Tomer Ziv-Baran, Gal Dubnov-Raz

**Affiliations:** 1 Faculty of Medicine, Tel Aviv University, Tel Aviv 69978, Israel; 2 Pediatric Exercise and Lifestyle Clinic, Safra Children’s Hospital, Sheba Medical Center, Tel HaShomer, Ramat Gan 52621, Israel

**Keywords:** adiposity, overweight, pediatric obesity, telemedicine, weight loss

## Abstract

The recommended treatment for children with obesity includes numerous consultations by a multidisciplinary team, which is very cumbersome. Telehealth can assist in administering frequent care to children with obesity, yet the exact approaches and modes of delivery are still explored. During the COVID-19 pandemic, we developed an intensive telehealth-based treatment program that included a rewarding app for children with obesity. The aim of this study was to compare 6-month changes in body mass index (BMI) and body fat percent between participants in the program (*n* = 70) vs. children that underwent historic on-site care (*n* = 87). After 6 months, more participants in the telehealth group continued treatment compared to the on-site group (79% vs. 60%, *p* < 0.001). A significant reduction in the median BMI z-score (zBMI) was seen after 6 months in both groups (*p* < 0.01), with a similar proportion of zBMI reductions (71% in the telehealth group, 75% in the comparison group, *p* = 0.76). No statistically significant differences were found between the study groups in 6-month changes in BMI, zBMI, body fat percent or fat z-scores. We conclude that our telehealth program, which was executed during the COVID-19 pandemic, resulted in a high proportion of children with zBMI reduction that was comparable with the more personal on-site care.

## 1. Introduction

The global number of children and adolescents with obesity increased 11-fold over the passing decades, from 11 million in 1975 to 124 million in 2016 [1]. The World Obesity Federation predicted that this number would continue to rise, from 158 million in 2020 to 258 million by 2030 [2]. The medical burden of both pediatric and adulthood obesity is well-known and includes numerous psychological and medical complications [3,4]. Treating children with obesity is, hence, essential for both body and mind, present and future.

Unfortunately, the current treatment offered to children with obesity is infrequently successful. Several meta-analyses from recent years have shown that mean body mass index (BMI) changes following usual treatment programs are small, and far from reaching clinically relevant magnitudes [5,6,7]. One of the reasons for this disappointing failure is that effective pediatric obesity treatment mandates numerous clinic visits over a long period of time. A US Preventive Services Task Force Recommendation Statement identified that, over 26 (!) contact hours are needed for improvements in weight status in children with obesity [8]. This large number of in-office visits is very difficult to maintain by parents and children for the months or years needed for proper treatment, given the repeated need to travel to clinics or after-school programs and the cumulative high cost. Therefore, the huge and growing burden of pediatric obesity collides with, and is perhaps fueled by, the difficulty of providing adequate, comprehensive long-term treatment.

During the COVID-19 pandemic and its associated lockdowns, many children’s already-unhealthy lifestyle habits deteriorated even further, with markedly decreased physical activity, increased screen time, and unhealthful diet practices [9,10,11], with subsequent increased obesity rates [12,13,14,15]. As in-office visits were minimized during the pandemic and telehealth was largely preferred and accepted by the public, the need and possibility for remote pediatric obesity treatment simultaneously emerged. Telehealth-based consultations for children with obesity were used before the pandemic, but quite uncommonly. In one review on this topic, only nine studies that used videoconferencing were identified, of which only three compared it to usual care [16]. Another review that compared telehealth interventions with face-to-face visits identified 10 such studies and concluded that most found no significant difference regarding BMI [17]. However, most of the older studies used one/two caretakers and relatively infrequent contact with the patients, some utilized only telephone messages/calls, and some did not offer direct comparisons between the treatment methods.

During the year 2020 and the COVID-19 pandemic, we established an intensive, multidisciplinary telehealth-based treatment program that included a rewarding smartphone app for children with obesity, as elaborated below. We are unaware of similar multidisciplinary pediatric obesity online treatment programs that utilized incentives via a rewarding app. The main aim of this study was to examine the effect of this program on measures of adiposity compared with historical, pre-COVID on-site care. While most previous studies showed that the results between telehealth and on-site care were comparable, our program had higher contact and volume than most published work [16,17], and we hypothesized that the magnitude of weight reduction would be equivalent or even larger with telehealth use.

## 2. Materials and Methods

### 2.1. Study Design

This was a historical cohort study conducted at the Pediatric Exercise and Lifestyle Clinic of the Edmond and Lily Safra Children’s Hospital, Sheba Medical Center, which is a large tertiary care center located in Central Israel. Electronic medical records of all children and adolescents with a listed diagnosis of obesity who visited the clinic from January 2014 to August 2022 were reviewed. The study was conducted according to the guidelines of the Declaration of Helsinki, and data extraction from the charts was approved by the Institutional Review Board of Sheba Medical Center, #SMC-21-8876, approved on 23 March 2022 and extended on 22 March 2023.

### 2.2. Study Population

The exposed group (telehealth group) included all patients who participated for at least 3 months in our telehealth-based treatment program conducted between 2020 and 2022. The inclusion criteria for the program included 70 males and females aged 10–18 years, obesity (defined as BMI ≥ 2 standard deviations according to the WHO growth reference [18]), and ownership of a smartphone. There were relatively few exclusion criteria in order to best reflect the whole population of children treated in our clinic and increase external validity. These included having significant physical or mental barriers to physical activity and/or to dietary changes, such as autistic spectrum disorder that substantially precluded dietary changes or sufficient exercise, significant congenital heart disease, or concurrent cancer treatment. Therefore, none of the participants was receiving medications known to cause weight gain (such as antipsychotics) and none was receiving weight-loss medications (such as metformin or liraglutide). The recruitment period lasted from February 2021 to February 2022, until 70 patients who met the inclusion and exclusion criteria were enrolled.

The non-exposed group (comparison group) included patients from both sexes aged 10–18 years (matching the age range in the telehealth group) who underwent on-site treatment during 2014–2019 for at least 3 months and had anthropometric data recorded at the time point of 3 and/or 6 months after their initial visit. The treatment in our clinic at that time included only a pediatric dietitian and a pediatrician trained in obesity and sports and exercise medicine. Patients were given dietary and exercise counseling and were invited for follow-up visits as needed, mostly every 3–4 weeks. Those who were compliant with the recommendations and continuously improved their weight status were usually invited in lower frequencies, usually every 2–3 months. Patients that were specifically preparing for bariatric surgery were excluded from the comparison group, as they received much more intensive treatment and evaluation than our usual population. As done for the telehealth group, patients with significant physical or mental barriers to physical activity and/or to dietary changes were also excluded. None of the participants were receiving medications known to cause weight gain or weight-loss medications, as described above.

### 2.3. Study Outcomes

The primary study outcome was the change in BMI standard deviation scores (z-score, zBMI) after 6 months in each of the study groups. Secondary outcomes were changes in body composition in both groups and changes in serum cardiometabolic risk factors after 6 months in the telehealth group.

### 2.4. The Telehealth Program

The telehealth program developed in our clinic during 2020 included 30 consultations for each participant over a period of 6 months: 3 physician appointments, 7 exercise consultations by an exercise physiologist, 10 dietary consultations by a pediatric dietitian, and 10 psychologist consultations to assist with goal setting and overall well-being. Three visits were conducted on-site for physician assessment, smartphone technical assistance, and physical measurements (baseline, at 3 months, and at the end of the 6-month period). All other consultations were 30-minute video calls that were distributed throughout the 6 months about once a week in order to provide frequent patient contact with at least one of the caretakers.

To increase adherence, participants in the program had the YuviTal app (YuviTal Health Ltd., Israel—https://yuvital.com/, last accessed on 26 June 2023) installed on their smartphone by our staff. This app monitors sleep, heart rate (via an activity tracker), and step counts, which were converted by the users to digital health tokens redeemed for actual prizes that were sent to their homes. We modified the app to be suitable for children by removing calorie counts and food rewards and tailored the prizes to include exercise equipment (e.g., flexible bands and water bottles), Bluetooth devices (e.g., headphones), or stationery equipment for school. Participants were given Garmin Vivosmart 4 wristbands or Apple watches (to Apple iPhone owners) for activity monitoring, as some schools and sports activities preclude the child from carrying a smartphone. One health token was given for each 1000 steps accumulated, and different amounts of tokens were needed for different prizes. The financial conversion rate for this project was ultimately ~25,000 steps to USD 1. Children were not limited to the number of prizes that they could purchase during the program.

### 2.5. Variables

Height was measured in each clinic visit, using the Seca 206 wall-mounted stadiometer (Seca gmbh, Hamburg, Germany), to the nearest 0.1 cm. Weight, fat mass, and fat-free mass were measured using the Tanita BC418-MA bio-impedance analyzer (Tanita Corporation, Tokyo, Japan) to the nearest 0.1 kg and 0.1 body fat percentage. Of note, this specific model has a very high agreement with dual-energy X-ray absorptiometry in body fat percent measurements, and it previously served to create body fat reference curves for children and adolescents [19]. BMI was calculated as weight in kg divided by the square of height in meters and converted to WHO growth reference age- and sex-appropriate z-scores [18], using the lmsGrowth Microsoft Excel add-in (Harlow Healthcare, South Shields, UK). Fat precent z-scores were also calculated using this program.

As part of their medical analysis at baseline, participants of the telehealth program were requested to provide blood test results of fasting plasms glucose, hemoglobin A1c, hepatocellular enzymes, and lipid profiles obtained by their healthcare medical provider. At the end of the program, we recommended repeating the tests, especially for children with abnormal baseline values. Both baseline and final blood tests were not mandatory, as the main program goal was to exert improvements in lifestyle in a positive atmosphere, with zBMI and body fat percent acting as markers for such improvements. Children who refused to undergo the blood tests were not obligated to do so.

### 2.6. Statistical Analyses

Descriptive statistics with frequency distributions of patient characteristics are presented as continuous and categorical variables, as appropriate. The distribution of continuous variables was evaluated using histograms and Q-Q plots and is presented as the mean ± standard deviation for the reader’s ease. Individual zBMI changes in both study groups are also presented [20]. Baseline characteristics and pre–post changes were compared between groups, using the Mann–Whitey U test, *t*-test, and chi square test, as applicable. The pre–post changes in zBMI within each group and in the cardiometabolic risk factors were examined using the Wilcoxon signed-ranks test. As a sensitivity analysis, we used the last-observation-carried-forward (LOCF) method, utilizing the 3-month data for imputing missing 6-month data of the dropouts. Multivariable logistic regression was used to examine the association between grouping and zBMI reduction, examined as either any decline or that greater than 0.2 and 0.25, which indicate clinical significance [8], while controlling for participant age. A two-sided *p*-value < 0.05 was considered to be statistical significance for all analyses, which were performed using IBM-SPSS version 28.

## 3. Results

Table 1 lists the clinical characteristics of the patients in each study group. The participants in the comparison group were older on average and had higher mean weights and BMI, yet their zBMI and body fat percent did not differ significantly from those of the telehealth group.

Of the 70 patients that began the telehealth program, 64 (91%) continued for at least 3 months, and 55 (79%) completed the 6-month period. The comparison group included 87 participants that had at least 3 months of data in their medical charts, and 52 participants had 6 months of data. The proportion of participants that had 6 months of data was significantly higher in the telehealth group (86% vs. 61%, *p* < 0.001). There were no significant differences in age, baseline zBMI, or zBMI at 3 months between participants with or without 6 months of data in either of the study groups.

The data extracted from the YuviTal app revealed a mean daily step count of 7520 ± 2749 for all participants, with no significant difference between the first and last months (7759 ± 3006 vs. 7430 ± 2420 steps/day, *p* = 0.136). The median purchase of prizes per participant over the program duration was 4, with an inter-quartile range of 0–8 and a range of 0–22.

**Figure 1:**
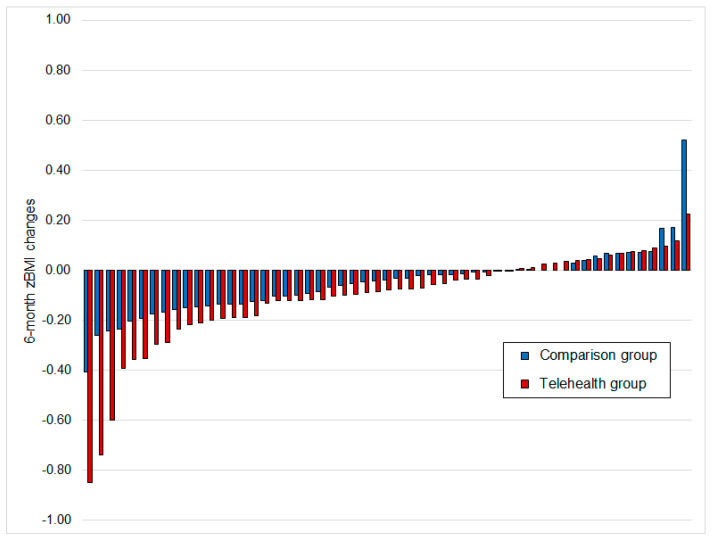
Individual zBMI changes after 6 months of treatment in both study groups.

In both groups, a significant reduction in the median zBMI was seen after 6 months (*p* < 0.01). Figure 1 presents the individual zBMI changes in both study groups.

Table 2 presents the changes in BMI and fat percent after 6 months in both study groups, both listed with the observed and LOCF values. There were no statistically significant differences between the study groups in any of the comparison methods of BMI and body fat percent. Of note, the proportion of zBMI reductions of over 0.2 and 0.25 were twice-to-over-three-fold higher in the telehealth group in both comparison methods (observed/LOCF), yet with no/borderline statistical significance attributed to the small numbers of patients in each group.

After employing regression analyses and adjusting for participant age, no significant difference between groups was observed in the odds for zBMI reduction in the telehealth vs. the comparison group (odds ratio (OR) = 0.8, 95% confidence interval (CI) 0.3–1.9, *p* = 0.54), zBMI reduction of >0.2 (OR = 2.3, 95%CI 0.7–7.4, *p* = 0.18), or of >0.25 (OR = 3.4, 95%CI 0.7–17.8, *p* = 0.14). Using LOCF imputations, similar associations were observed, and no significant differences were found.

Table 3 presents the changes in serum cardiometabolic risk factors in the telehealth group. The only statistically significant change seen was in hemoglobin A1c, which increased in a clinically negligible magnitude.

## 4. Discussion

The aim of this study was to compare the changes in BMI and body fat after 6 months of participation in an intensive telehealth-based treatment program for children with obesity executed during the COVID-19 pandemic, with historical, pre-COVID on-site care with visits scheduled as needed. A significant improvement in body measures was seen in both groups, with over 70% of patients with available 6 months of data demonstrating reduced zBMI. Somewhat to our disappointment, yet in accordance with previous research, there were no statistically significant between-group differences in measures of adiposity. An encouraging finding was the much higher proportion of patients with clinically significant zBMI reductions of >0.2 and >0.25, albeit in small absolute numbers.

This study was performed to complement previous work in the field of telehealth-based pediatric obesity care by examining the effect of an intensive, high-frequency, multidisciplinary treatment program reinforced with a rewarding app. Only a few published studies have examined multidisciplinary pediatric obesity treatment programs that used video conversations. Irby et al. [21] placed videoconferencing monitors in pediatric offices in 2009 in order to overcome the geographical barrier for receiving multidisciplinary obesity care in North Carolina, USA. Among the 35 children who participated, a high rate of zBMI reduction of 64% was seen; however, it did not differ significantly from the 69% seen with in-office visits, similar to our findings. Nevertheless, a decreased attrition rate was seen in rural patients after implementing telemedicine care, as we encountered as well. Slusser et al. [22] reviewed charts of 62 low-income underinsured children with obesity managed by a multidisciplinary team, using telehealth, during 2011–2014 in the Los Angeles area. Among the 32 patients with data, 41% reduced their zBMI. Reschke et al. [23] utilized videoconferencing during the first COVID-19 lockdowns as an alternative to coming to the clinic, thus enabling their patients to continue effective treatment and improvement of their weight status, as we also believe was the major effect of our program in those times.

Telehealth has an additional important benefit to those listed above, which is high parent and patient satisfaction. One review of various telehealth methods (SMS, email, social media, video calls, phone calls, web-based systems, or apps) found that most parents had an overall positive experience with using digital health technology in pediatric overweight and obesity [24], reporting that they saved time and money on travel and found it to be generally convenient. A survey of caretaker opinions regarding telehealth conducted during the COVID-19 pandemic listed quality communication with physicians, savings in travel time, and cost-effectiveness, yet identified challenges such as lack of in-person interaction (as we also certainly felt in the clinic), fear of compromised confidentiality, and the potential for misdiagnosis [25]. Collectively, telehealth in obesity treatment is clinically useful and has its benefits, but it still requires some refining.

Perhaps the major downside to frequent telehealth visits and the technology used is its high cost. Tully et al. [26] calculated that their digital pediatric weight-management program from Ireland, delivered via a smartphone app, cost over X5 more than usual care. We did not perform a formal cost analysis per patient, but we can calculate that the cost of the 30 clinic visits and the app use in the telehealth program is about X3 that of six monthly on-site visits with a dietitian and physician. It should be noted that the recent American Academy of Pediatrics guidelines regarding pediatric obesity recommend intensive health behavior as the foundational approach to achieve body mass reduction or the attenuation of excessive weight gain in children, administered to patient and family by a multidisciplinary treatment team [3]. Hence, we should aspire to provide such comprehensive care both face-to-face and online as well. However, the high cost and need for frequent contact are not practical for all families, and, indeed, there are many publications of “thinner“ telehealth programs that included only one or two caretakers, or even simply phone calls/text messages in older work, that provided comparable results to on-site [16,17,27]—as we found also. One study, for example, that compared group videoconferencing with telephone calls only found comparable results in mean zBMI, diet, physical activity, or quality of life [28]. Collectively, it appears that the optimal “dose” of tele-visits and their modes of delivery are yet to be identified. Our clinical experience coincides with the published data in these reviews, indicating that there is no “one frequency fits all” regarding pediatric obesity care. Some families need more contact and more caretakers, whereas others can be managed with mainly a dietitian and less frequent visits/video calls. Furthermore, one important study conducted during the COVID-19 pandemic in July–December 2020 found that in-person clinic visits for pediatric obesity treatment were highly preferred: 72% of visits were on-site vs. 28% that used telehealth [29]. Telehealth was selected more often for follow-up visits compared with initial visits, just as we felt as well. Moreover, another downside of video chats was the difficulty to create a person-to-person relationship, as stated above [25]. We, hence, recommend that specialist pediatric obesity clinics be able to provide all forms and levels of care by having all clinical personnel needed, with face-to-face, video chats, apps, or automated reminders/messages options in their toolbox, tailored to each family’s needs and preferences.

The notion of using a rewarding app for increasing activity in our population originated from studies in adults with overweight, showing that financial incentives can assist in weight loss [30,31] and that gamification with financial incentives can increase activity [32]—though generally with small effects. We attempted to disconnect rewards and prizes from weight loss, as we believe that weight change should not be a direct goal set in front of the child, and we focused on rewarding exercise. We were pleased to see that, overall, the children accumulated a fair number of daily steps and that this effect did not wane during the program. Two previous studies also attempted to increase activity in children through incentives. In a study from Singapore, Finkelstein et al. [33] offered toy-store vouchers to 6–12-year-olds who accumulated ≥8000 steps per day for over one half of the month, with additional prizes offered through lotteries. There was no dietary intervention, and there was no significant effect on BMI, fitness, or quality of life. Another study, in native American adolescents with overweight, also used financial incentives for exercise in the form of direct payment with no dietary treatment and found a modest effect on increased activity, again with no significant effect on BMI [34]. Another possible benefit of exergaming/rewarding programs is a reduced dropout rate, as seen in our comparison. Previous evidence does support the role of financial incentives in reducing attrition and increasing participation in adult programs related to weight loss [31].

We acknowledge that our study has limitations. Firstly, as this was not a controlled interventional trial, there were some between-group differences in baseline parameters, as the comparison group was older on average and, thus, could have had more pubertal participants. This can explain their higher mean weight and BMI, but since zBMI and body fat were comparable between groups, this age and weight difference was overcome. In addition, not all patients in the historic comparison group had measurements exactly at the 3/6-month time point to be available for analysis. Second, we did not have data on step counts or cardiovascular risk factors in the comparison group and, thus, were limited to a comparison of body measures only. Thirdly, the telehealth program was implemented during the 2020–2022 COVID-19 period, which does not reflect “normal lives”. On the one hand, children and parents were highly available for video calls during the day and had more time to exercise. On the other, the lack of constructed school and workdays resulted in the well-known increases in sedentary times and unhealthful diet practices [35]. This was a challenging environment for the children with obesity treated in our program at the time, during which we are able to obtain relatively high rates of adherence and improvements in body measures though the telehealth program.

Our study strengths are incorporating a structured program that was more intensive than most previous work, of 30 visits over 6 months, for a total of ~15 contact hours, thus adding significantly to the available literature; inclusion of a rewarding app specifically tailored for children; comparison to a face-to-face group; and including children from the general population of Central Israel, as opposed to low-income rural patients, which were the focus of most previous telehealth publications.

## 5. Conclusions

In conclusion, this multidisciplinary treatment program with a rewarding app for children with obesity produced comparable results to the more personal on-site care, and with lower attrition. Future studies of telehealth care in pediatric obesity should focus on identifying the best modes of treatment and its most important components in order to minimize the costs and treatment burden for the families. In addition, randomized controlled trials outside the COVID pandemic, in current “real life”, should also be performed.

## Figures and Tables

**Table 1:** Characteristics of the study participants in both study groups. Data are presented as the mean ± standard deviation or *n* (%).

Characteristic	Comparison Group	Telehealth Group	*p*-Value
Age (years)	14.1 ± 2.1	12.9 ± 1.75	<0.001
Males (*n*, %)	46 (53%)	35 (55%)	0.825
Height (cm)	161.2 ± 10.9	160.0 ± 11.2	0.212
Weight (kg)	86.6 ± 23.4	77.1 ± 19.7	0.04
BMI (kg/m^2^)	32.9 ± 7.0	29.7 ± 4.7	0.008
zBMI	2.12 ± 0.49	2.03 ± 0.42	0.124
Fat percent (%)	39.0 ± 8.1	38.3 ± 8.0	0.625
z fat percent	2.55 ± 0.68	2.34 ± 0.62	0.063

**Table 2:** Changes in BMI and fat percent after 6 months in both study groups. Data are presented as the mean ± standard deviation or proportions, as appropriate.

Characteristic	Comparison Group—Observed (*n* = 52)	Telehealth Group—Observed (*n* = 55)	*p*-Value	Comparison Group— LOCF (*n* = 87)	Telehealth Group— LOCF (*n* = 64)	*p*-Value
BMI change (kg/m^2^)	−0.20 ± 1.38	−0.25 ± 1.80	0.876	−0.21 ± 1.26	−0.19 ± 1.72	0.923
Reduced BMI (*n*, %)	27 (52%)	31 (56%)	0.572	45 (52%)	35 (55%)	0.718
zBMI change	−0.011 ± 0.337	−0.113 ± 0.198	0.298	−0.022 ± 0.271	−0.099 ± 0.189	0.234
Reduced zBMI (*n*, %)	39 (75%)	39 (71%)	0.756	60 (69%)	44 (69%)	0.977
Reduced zBMI > 0.2	5 (9.6%)	11 (20.0%)	0.122	8 (9.2%)	11 (17.2%)	0.143
Reduced zBMI > 0.25	2 (3.8%)	8 (14.5%)	0.094	3 (3.4%)	8 (12.5%)	0.054
Fat percent change (%)	−0.85 ± 3.37	−1.05 ± 3.33	0.774	−0.63 ± 3.08	−0.76 ± 3.28	0.817
Reduced fat percent (*n*, %)	29 (56%)	34 (62%)	0.863	43 (49%)	38 (59%)	0.226
z fat percent change	−0.07 ± 0.32	−0.06 ± 0.29	0.856	−0.05 ± 0.30	−0.04 ± 0.28	0.768
Reduced z fat precent (*n*, %)	25 (48%)	32 (58%)	0.401	40 (46%)	35 (55%)	0.290

BMI—body mass index; zBMI—BMI z-score; LOCF—last observation carried forward.

**Table 3:** Changes in serum cardiometabolic risk factors in the telehealth group. Values are presented as the mean ± standard deviation.

	Glucose	Hemoglobin A1c	ALT	Triglycerides	LDL	HDL
Baseline	89.6 ± 7.0	5.2 ± 0.4	26.6 ± 24.4	98.5 ± 39.9	90.9 ± 23.8	46.8 ± 9.1
End	92.2 ± 7.7	5.3 ± 0.3	23.3 ± 24.3	95.2 ± 48.6	96.3 ± 24.3	47.1 ± 7.6
Change	−1.8 ± 6.9	−0.1 ± 0.2	0.7 ± 8.2	−5.9 ± 44.8	−2.5 ± 16.8	0.9 ± 5.7
*p*-Value	0.122	0.044	0.269	0.910	0.388	0.527

Baseline values are from all children that performed baseline blood tests (*n* = 63). Changes were calculated and compared only within the group of children that also performed the final blood tests (*n* = 40 for glucose, *n* = 20 for hemoglobin A1c, *n* = 38 for ALT, *n* = 37 for triglycerides, *n* = 34 for LDL, and *n* = 36 for HDL). ALT = alanine aminotransferase; LDL = low-density lipoprotein; HDL = high-density lipoprotein.

## Data Availability

The data presented in this study are available upon request from the corresponding author.

## References

[1] NCD Risk Factor Collaboration (NCD-RisC) (2017). Worldwide trends in body-mass index, underweight, overweight, and obesity from 1975 to 2016: A pooled analysis of 2416 population-based measurement studies in 128.9 million children, adolescents, and adults. Lancet.

[2] Lobstein T., Jackson-Leach R. (2016). Planning for the worst: Estimates of obesity and comorbidities in school-age children in 2025. Pediatr. Obes..

[3] Hampl S.E., Hassink S.G., Skinner A.C., Armstrong S.C., Barlow S.E., Bolling C.F., Avila Edwards K.C., Eneli I., Hamre R., Joseph M.M. (2023). Clinical Practice Guideline for the Evaluation and Treatment of Children and Adolescents with Obesity. Pediatrics.

[4] GBD 2015 Obesity Collaborators (2017). Health effects of overweight and obesity in 195 countries over 25 years. N. Engl. J. Med..

[5] Kobes A., Kretschmer T., Timmerman G., Schreuder P. (2018). Interventions aimed at preventing and reducing overweight/obesity among children and adolescents: A meta-synthesis. Obes. Rev..

[6] Ells L.J., Rees K., Brown T., Mead E., Al-Khudairy L., Azevedo L., McGeechan G.J., Baur L., Loveman E., Clements H. (2018). Interventions for treating children and adolescents with overweight and obesity: An overview of Cochrane reviews. Int. J. Obes..

[7] Gates A., Elliott S.A., Shulhan-Kilroy J., Ball G.D.C., Hartling L. (2020). Effectiveness and safety of interventions to manage childhood overweight and obesity: An Overview of Cochrane systematic reviews. Paediatr. Child Health.

[8] Grossman D.C., Bibbins-Domingo K., Curry S.J., Barry M.J., Davidson K.W., Doubeni C.A., Epling J.W., Kemper A.R., Krist A.H., US Preventive Services Task Force (2017). Screening for Obesity in Children and Adolescents: US Preventive Services Task Force Recommendation Statement. JAMA.

[9] Pietrobelli A., Pecoraro L., Ferruzzi A., Heo M., Faith M., Zoller T., Antoniazzi F., Piacentini G., Fearnbach S.N., Heymsfield S.B. (2020). Effects of COVID-19 Lockdown on Lifestyle Behaviors in Children with Obesity Living in Verona, Italy: A Longitudinal Study. Obesity.

[10] Ruíz-Roso M.B., de Carvalho Padilha P., Matilla-Escalante D.C., Brun P., Ulloa N., Acevedo-Correa D., Ferreira Peres W.A., Martorell M., Bousquet Carrilho T.R., De Oliveira Cardoso L. (2020). Changes of physical activity and ultra-processed food consumption in adolescents from different countries during COVID-19 pandemic: An observational study. Nutrients.

[11] Neshteruk C.D., Zizzi A., Suarez L., Erickson E., Kraus W.E., Li J.S., Skinner A.C., Story M., Zucker N., Armstrong S.C. (2021). Weight-Related Behaviors of Children with Obesity during the COVID-19 Pandemic. Child. Obes..

[12] Kim E.S., Kwon Y., Choe Y.H., Kim M.J. (2021). COVID-19-related school closing aggravate obesity and glucose intolerance in pediatric patients with obesity. Sci. Rep..

[13] Jenssen B.P., Kelly M.K., Powell M., Bouchelle Z., Mayne S.L., Fiks A.G. (2021). COVID-19 and Changes in Child Obesity. Pediatrics.

[14] Dubnov-Raz G., Maor S., Ziv-Baran T. (2022). Pediatric obesity and body weight following the COVID-19 pandemic. Child Care Health Dev..

[15] Anderson L.N., Yoshida-Montezuma Y., Dewart N., Jalil E., Khattar J., De Rubeis V., Carsley S., Griffith L.E., Mbuagbaw L. (2023). Obesity and weight change during the COVID-19 pandemic in children and adults: A systematic review and meta-analysis. Obes. Rev..

[16] Moorman E.L., Koskela-Staples N.C., Mathai B.B., Fedele D.A., Janicke D.M. (2021). Pediatric Obesity Treatment via Telehealth: Current Evidence and Future Directions. Curr. Obes. Rep..

[17] Whitley A., Yahia N. (2021). Efficacy of Clinic-Based Telehealth vs. Face-to-Face Interventions for Obesity Treatment in Children and Adolescents in the United States and Canada: A Systematic Review. Child. Obes..

[18] World Health Organization Growth Reference Data for 5–19 Years. BMI-for-Age. https://www.who.int/tools/growth-reference-data-for-5to19-years/indicators/bmi-for-age.

[19] McCarthy H.D., Cole T.J., Fry T., Jebb S.A., Prentice A.M. (2006). Body fat reference curves for children. Int. J. Obes..

[20] Dubnov-Raz G., Berry E.M. (2017). What paediatric obesity treatment programmes work, and how can we measure their success?. Acta Paediatr..

[21] Irby M.B., Boles K.A., Jordan C., Skelton J.A. (2012). TeleFIT: Adapting a Multidisciplinary, Tertiary-Care Pediatric Obesity Clinic to Rural Populations. Telemed. e-Health.

[22] Slusser W., Whitley M., Izadpanah N., Kim S.L., Ponturo D. (2016). Multidisciplinary Pediatric Obesity Clinic via Telemedicine within the Los Angeles Metropolitan Area. Clin. Pediatr..

[23] Reschke F., Galuschka L., Landsberg S., Weiner C., Guntermann C., Sadeghian E., Lange K., Danne T. (2022). Successful telehealth transformation of a pediatric outpatient obesity teaching program due to the COVID-19 pandemic—The “Video KiCK” program. J. Pediatr. Endocrinol. Metab..

[24] Fidjeland T.G., Øen K.G. (2022). Parents’ Experiences Using Digital Health Technologies in Paediatric Overweight and Obesity Support: An Integrative Review. Int. J. Environ. Res. Public Health.

[25] Kodjebacheva G.D., Tang C., Groesbeck F., Walker L., Woodworth J., Schindler-Ruwisch J. (2023). Telehealth Use in Pediatric Care during the COVID-19 Pandemic: A Qualitative Study on the Perspectives of Caregivers. Children.

[26] Tully L., Sorensen J., O’Malley G. (2021). Pediatric Weight Management Through mHealth Compared to Face-to-Face Care: Cost Analysis of a Randomized Control Trial. JMIR mHealth uHealth.

[27] Azevedo L.B., Stephenson J., Ells L., Adu-Ntiamoah S., DeSmet A., Giles E.L., Haste A., O’Malley C., Jones D., Chai L.K. (2022). The effectiveness of e-health interventions for the treatment of overweight or obesity in children and adolescents: A systematic review and meta-analysis. Obes. Rev..

[28] Davis A.M., Sampilo M., Gallagher K.S., Dean K., Saroja M.B., Yu Q., He J., Sporn N. (2016). Treating rural paediatric obesity through telemedicine vs. telephone: Outcomes from a cluster randomized controlled trial. J. Telemed. Telecare.

[29] Siegel R., Stackpole K., Kirk S., Kharofa R. (2022). Families Chose In-Person Visits over Telehealth for Pediatric Weight Management during the COVID-19 Pandemic. Child. Obes..

[30] Paul-Ebhohimhen V., Avenell A. (2008). Systematic review of the use of financial incentives in treatments for obesity and overweight. Obes. Rev..

[31] Ananthapavan J., Peeterson A., Sacks G. (2018). Paying people to lose weight: The effectiveness of financial incentives provided by health insurers for the prevention and management of overweight and obesity—A systematic review. Obes. Rev..

[32] Agarwal A.K., Waddell K.J., Small D.S., Evans C., Harrington T.O., Djaraher R., Oon A.L., Patel M.S. (2021). Effect of Gamification with and without Financial Incentives to Increase Physical Activity among Veterans Classified as Having Obesity or Overweight: A randomized clinical trial. JAMA Netw. Open.

[33] Finkelstein E.A., Tan Y.-T., Malhotra R., Lee C.-F., Goh S.-S., Saw S.-M. (2013). A Cluster Randomized Controlled Trial of an Incentive-Based Outdoor Physical Activity Program. J. Pediatr..

[34] Short K.R., Chadwick J.Q., Cannady T.K., Branam D.E., Wharton D.F., Tullier M.A., Thompson D.M., Copeland K.C. (2018). Using financial incentives to promote physical activity in American Indian adolescents: A randomized controlled trial. PLoS ONE.

[35] Karatzi K., Poulia K.-A., Papakonstantinou E., Zampelas A. (2021). The Impact of Nutritional and Lifestyle Changes on Body Weight, Body Composition and Cardiometabolic Risk Factors in Children and Adolescents during the Pandemic of COVID-19: A Systematic Review. Children.

